# Immune system function, stress, exercise and nutrition profile can affect pregnancy outcome: Lessons from a Mediterranean cohort

**DOI:** 10.3892/etm.2012.849

**Published:** 2012-12-04

**Authors:** D. MPARMPAKAS, A. GOUMENOU, E. ZACHARIADES, G. PADOS, Y. GIDRON, E. KARTERIS

**Affiliations:** 1 Centre for Cell and Chromosome Biology, Division of Biosciences, Brunel University, Uxbridge, UK;; 2 Department of Obstetrics and Gynecology, University Hospital, University of Crete Medical School, Heraklion, Crete;; 3 University of Thessaloniki Medical School, Thessaloniki, Greece;; 4 Free University of Brussels (VUB), Brussels, Belgium

**Keywords:** maternal stress, smoking, birth weight

## Abstract

Pregnancy is associated with major physiological and future psychosocial changes, and maternal adaptation to these changes is crucial for normal foetal development. Psychological stress in pregnancy predicts an earlier birth and lower birth weight. Pregnancy-specific stress contributes directly to preterm delivery. The importance of nutrition and exercise during pregnancy with regard to pregnancy outcome has long been acknowledged. This importance has only been further emphasized by the recent changes in food quality and availability, lifestyle changes and a new understanding of foetal programming’s effects on adult outcomes. We hypothesised that for a successful pregnancy certain events at a nutritional, immune, psycho-emotional and genetic level should be tightly linked. Therefore, in this study we followed an ‘integrative’ approach to investigate how maternal stress, nutrition, pregnancy planning and exercise influence pregnancy outcome. A key finding of our study is that there was a significant reduction in the intake of alcohol, caffeine-containing and sugary drinks during pregnancy. However, passive smoking in the household remained unchanged. In terms of immune profile, a significant inverse correlation was noted between difficulty to ‘fight’ an infection and number of colds (r=−0.289, P=0.003) as well as the number of infections (r=−0.446, P<0.0001) during pregnancy. The vast majority of the pregnant women acquired a more sedentary lifestyle in the third trimester. In planned, but not in unplanned, pregnancies stress predicted infant weight, independent of age and body mass index (BMI). Notably, in mothers with negative attitudes towards the pregnancy, those with an unplanned pregnancy gave birth to infants with significantly higher weights than those with planned pregnancies. Collectively these data suggest that there is a higher order of complexity, possibly involving gene-environment interactions that work together to ensure a positive outcome for the mother as well as the foetus.

## Introduction

The importance of nutrition during pregnancy with regard to pregnancy outcome has long been acknowledged. This importance has only been further emphasized by the recent changes in food quality and availability, lifestyle changes and a new understanding of foetal programming’s effects on adult outcomes (1). The Mediterranean dietary pattern (MDP) exerts certain beneficial effects, since it appears to be associated with a reduction in the risk of offspring affected by spina bifida (2) and with children presenting as less wheezy or asthmatic (3). Recently, Mariscal-Arcas *et al*(4), proposed a diet quality index for pregnancy based on MDP evaluating the diet of a group of pregnant women by applying the Mediterranean Diet Score (MDS) and evaluating their intake of micronutrients required in optimal amounts during pregnancy.

Pregnancy is also associated with major physiological and future psychosocial changes, and maternal adaptation to these changes is crucial for normal foetal development. Psychological stress in pregnancy predicts an earlier birth and lower birth weight. Perceived life-event stress, as well as depression and anxiety, predicted lower birth weight, decreased Apgar scores and occurrence of babies small for their gestational age (5). Therefore, it appears that pregnancy-specific stress may be a more powerful contributor to birth outcomes than general stress (6).

It has also been reported that women who had unplanned pregnancies had more psychological problems throughout their pregnancies when compared with those that planned their pregnancy (7). These novel findings suggest an important finding, it is estimated that approximately 87 million pregnancies take place every year worldwide, out of which 41 million result in labour (WHO 2005). Our working hypothesis is that for a successful pregnancy certain events at the nutritional, immune, psycho-emotional and genetic level may be tightly linked. Therefore, in this study we followed a ‘holistic’ approach to investigate how maternal stress, nutrition, pregnancy planning and exercise influence pregnancy outcome.

## Subjects and methods

### Subjects

The study population consisted of pregnant women (n=113) attending the Department of Obstetrics and Gynaecology, University Hospital, University of Crete Medical School (Crete, Greece). The participants were in the third trimester of their pregnancy. All participants gave informed consent to participate in the study and ethical approval was granted by the local Ethics Committee of the Hospital.

### Construction of the questionnaire

A questionnaire was constructed comprising 59 questions, which were associated with the following specific subject areas and variables: i) body mass index (BMI), ii) immune problems, iii) exercise, iv) nutrition, v) stress and vi) medical history. Specific questions were used to acquire anthropometric data, which included gestational age, weight of the mother prior to conception as well as the weight of the mother at the time and self-reported height. Behavioural data that were collected, including smoking and drinking habits, and physical activity, the latter based on the measure of Paffenbarger *et al*(8). Also, multi-mineral/multi-vitamin supplement usage before and during pregnancy was assessed. There were specific questions associated with the stress profile, which included: whether the pregnancy was planned or not planned and how stressed each woman was during pregnancy, with responses ranging from 1 to 4 (1, low; 2, medium; 3, high; 4, very high). This stress questionnaire was adapted from a study by Wang *et al*(9), where women with dysmenorrhoea were asked to describe their stress in preceding cycles as ‘low’, ‘medium’ or ‘high’, whereas the questions associated with nutritional status were based on the Department of Health Services - Women, Infants and Children Program (CA, USA; www.sonoma-county.org/health/wic/en/applications/pregnancy.pdf).

### Statistical analysis

We tested the association between continuous predictors (e.g. age, stress levels) and infant weight using Pearson correlations and partial correlations (when adjusting for age and BMI). We used Student’s t-tests for dichotomous predictors (e.g. planned pregnancy) and analysis of variance (ANOVA) for testing the association between attitude type (initially in three categories) and infant birth weight. For the correlation studies, a two-tailed test using SPSS (version 18) was performed. P<0.05 was considered to indicate a statistically significant result.

## Results

### General profile of the participants of the study

The data of the general profile that were collected are presented in Table I. The majority of the participants appeared to be of normal BMI. Information of the weight of the mother as a newborn, as well as if she was born at term or pre-term, was also collected, and the majority of the participants were born in the normal for gestational age range and after the 37th week of gestation, which is considered to be at term. The maternal body shape was also recorded to understand the participant adiposity type. The majority of women reported to have a pear-shape body type. Information on the duration of the pregnancy as well as the weight of the foetus was also recorded. Notably, a slightly higher percentage of the infants were born prematurely (<37 weeks).

### Immune profile of the participants

The second profile that was analysed was the immune profile of the participants, which consisted of 7 questions in total (Table II). A significant inverse correlation was observed between difficulty to ‘fight’ an infection and number of colds (r=−0.289, P=0.003) as well as number of infections (r=−0.446, P<0.0001) during pregnancy. An inverse correlation was also observed between pregnancy days and number of infections during pregnancy (r=−0.212, P=0.004). Notably, the use of antibiotics was also inversely correlated with difficulty to get rid of a cold (r=−0.422, P<0.0001) and how prone these women were to cystitis infections (r=−0.389, P<0.0001).

### Exercise profile prior to and during pregnancy

The majority of the women reported to have light activity, this consisted of 2–4 flights of stairs that they climbed on a daily basis and this is consistent before as well as during pregnancy (Table III). As far as their walking habits were concerned, an almost equal percentage of women reported a light or moderate walking activity before pregnancy, whereas this moderate activity was lower during pregnancy and the light walking activity (equivalent of 2–4 city blocks) was the highest category. With regard to participation in light sports, the inactivity was increased to 60% during pregnancy. When the participants were asked the number of h/week they would participate in any strenuous sports, including running, cycling or tennis, again the majority reported to be inactive both before and during their pregnancy.

### Nutrition profile before and during pregnancy

**Figure 1.**
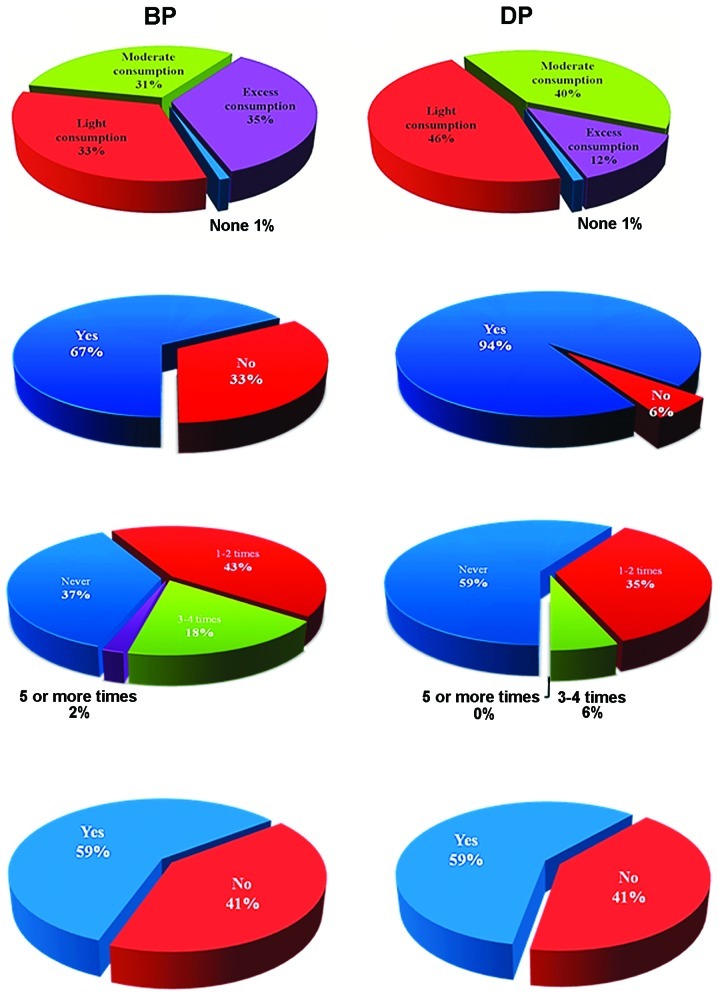
Nutrition profile before (BP) and during (DP) pregnancy. (A and B) Weekly consumption of foods that are high in lipids and more specifically in saturated lipids (e.g. pies, pastries, fried foods). (C and D) Daily consumption of iron-rich foods (e.g. lean red meat, chicken, green leafy vegetables). (E and F) Weekly consumption of fast-food. (G and H) Percentage of passive smoking in the household.

The vast majority of the participants were not vegetarians and this percentage remained unchanged during pregnancy (Table IV). When the participants were asked how many of their weekly meals included foods that are high in lipids and more specifically in saturated lipids (e.g. pies, pastries, fried foods) the majority reported to excessively (≥5 times/week) consume these types of foods rich in lipids before pregnancy, whereas this percentage was lowered significantly during pregnancy, where the majority reported light consumption of these foods (Fig. 1A and B). A high percentage of women reported to moderately consume vegetables on a daily basis before pregnancy and this was maintained during pregnancy. This was also consistent with the responses obtained with regard to the daily consumption of fruits, before as well as during pregnancy, where the majority reported to moderately consume fruits in both periods.

Also, the majority of the participants reported to eat iron-rich foods (e.g. lean red meat, chicken, green leafy vegetables) on a daily basis before pregnancy, but the percentage of pregnant women consuming more iron-rich foods on a daily basis was significantly increased compared to pre-pregnancy (Fig. 1C and D). A high percentage of women reported to consume 2–3 cups of coffee or other caffeine-containing beverages before pregnancy, however, during pregnancy the majority of women reduced their daily consumption to 1 cup or less. In addition, the percentage of women drinking 2–3 sugary drinks on a daily basis was reduced during pregnancy to 1 or fewer. The majority of participants reported not to consume any alcoholic beverages before or during pregnancy. Lastly, the majority of the participants reported to eat fast food 1–2 times/week whereas this was reduced to none during pregnancy (Fig. 1E and F).

With regard to smoking profile, the majority (79%) of women reported that they did not smoke immediately before their pregnancy and this was maintained during pregnancy. However, we emphasize that only 7% of the women reported to have stopped smoking while pregnant, whereas the remainder of the participants (14%) reported to have continued this habit. Notably, when asked if anyone in the household was smoking before and if they had continued to do so during pregnancy, a high percentage reported that, before as well as during pregnancy, they were exposed to passive smoking (Fig. 1G and H).

### Maternal stress and foetal birth weight

**Figure 2.**
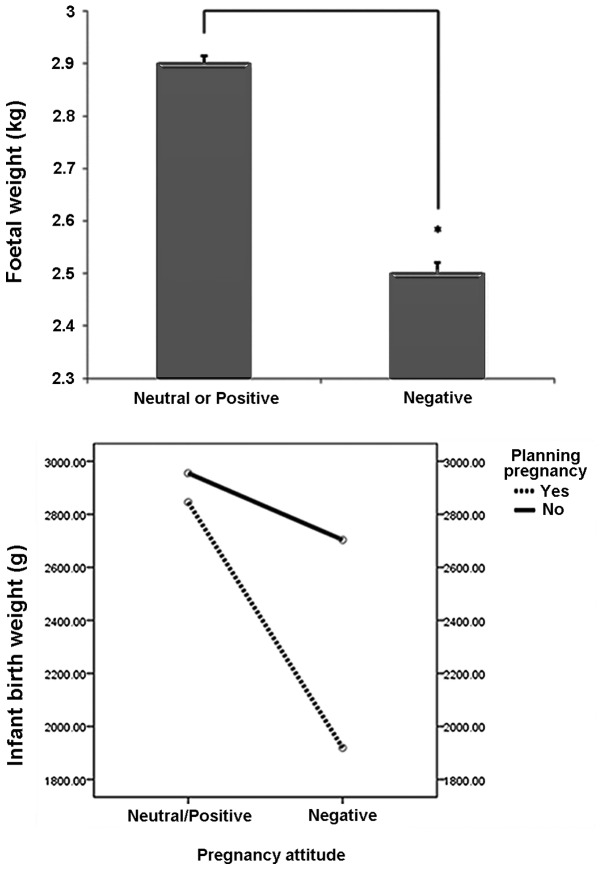
Maternal stress and foetal outcome. (A) Prenatal maternal attitude towards the pregnancy and foetal weight: neutral or positive and negative attitudes. (B) Effects of maternal attitude to pregnancy and pregnancy planning on infant birth weight.

Maternal attitudes were correlated with self-reported stress status ranging from low and medium (low stress response) to high and very high (high stress response). The source of the stress was not reported. The women with negative attitudes towards their pregnancy reported significantly higher levels of stress during pregnancy (2.8) compared to women with neutral (1.6) or positive attitudes towards their pregnancy [1.4; t(95)=9.8, P<0.001]. Since women with neutral and positive attitudes towards the pregnancy did not differ on stress levels [t(95)=1.0, P>0.05], and due to the small number of women with neutral attitudes, both groups were merged. The women forming the first group with the positive and neutral attitudes were compared with those with negative attitudes. The women with negative attitudes during pregnancy gave birth to infants with significantly lower birth weights (2.5 kg) than those with a positive or neutral attitude towards their pregnancy (2.9 kg; F(1,71)=4.2, P<0.05; Fig. 2A), independent of their age and BMI.

We then tested whether maternal background variables predicted infant birth weight. Age, BMI, physical activity, alcohol consumption and smoking habits during pregnancy were not associated with infant birth weight (all P>0.05). The majority of immune symptoms of women (i.e. difficulty overcoming an infection, number of colds or infections during pregnancy, inflammatory diseases, proneness to cystitis/thrush, allergies, use of antibiotics) were also not associated with infant birth weight (all P>0.05).

### Effects of maternal attitude to pregnancy and pregnancy planning on infant birth weight

In planned pregnancies, stress predicted infant weight, independent of age and BMI (r=−0.44, P=0.01), whereas in unplanned pregnancies stress did not predict infant weight (r=−0.19, P=0.23). In mothers with neutral or positive attitudes towards pregnancy, planning the pregnancy had no effect on infant birth weight [F(1,30)=0.091, not significant]. However, in mothers with negative attitudes towards the pregnancy, those with an unplanned pregnancy gave birth to infants with significantly higher weights (2681.9 g) than those with planned pregnancies [1917.8 g; F(1,36)=7.074, P=0.012; Fig. 2B].

## Discussion

The present study extends previous findings and provides new evidence on how psychosocial environment (i.e. stress/pregnancy planning) affects foetal outcome. We have shown that there was a significant reduction in the intake of alcohol, caffeine-containing and sugary drinks, as well as sugary refreshments during pregnancy. In our cohort 14% of women smoked during pregnancy. This is comparable to a recent study of Australian women that showed that 14.8% of non-indigenous women were smoking during pregnancy (10). Maternal smoking during pregnancy is a well-established risk factor for perinatal mortality, miscarriage and premature births (11) and exposure to heavy smoking *in utero* increases the risk of nicotine dependence in adulthood (10). In this study, another notable finding was identified, that parental/passive smoking was not reduced in the household during pregnancy, staying at a high rate of 59%. This could be detrimental, as all types of passive smoking have been associated with a significant increase in the risk of infants developing lower respiratory infections in the first two years of life (12).

In this questionnaire seven questions associated with the immune profile of this cohort were incorporated. We have done so, as acute infections in pregnant women are often associated with adverse effects, including miscarriage, preterm labour, preeclampsia (PE) or even stillbirth (13–15). Notably, a significant inverse correlation has been identified between difficulty to ‘fight’ an infection and number of colds and number of infections during pregnancy. Similar data have been obtained by a recent study of Australian women, where a cold was the most common infection reported using a similar self-reported method (16). In the study by Lain *et al*(16), only 21% of the women that reported an infection sought medical attention. We do not have such records for our cohort. However, our data on the immune profile also have certain strengths as it includes the investigation of numerous rather than a single infection and incorporates both chronic and acute infections.

With regard to the effect of caffeine during pregnancy, there is still some controversy in the field, as it has been implicated as a cause of spontaneous abortion, intrauterine growth restriction (IUGR), low birth weight and pre-term delivery (17). However, other investigators failed to find any association between caffeine intake and poor pregnancy outcomes (18). Adeney *et al*(19) revealed that moderate caffeine consumption during pregnancy exerts a protective effect towards gestational diabetes mellitus (GDM) (19). These mixed results may arise due to the problem of accurately assessing the caffeine intake. In addition, the amount of caffeine varies greatly in different coffee chains. In a recent study, caffeine levels varied up to 6-fold (20). In our cohort, a significant reduction in caffeine intake was noted, although we were not able to quantify the precise amount ingested. Nawrot *et al*(21) suggested that women of reproductive age should consume less than 300 mg of caffeine/day.

In this cohort, a significant decrease in the consumption of sugar-containing drinks during pregnancy was observed. In the USA for example, sugar-sweetened soft drinks are the principal energy contributors in the diet (22) and they appear to play a role in the obesity epidemic due to their high content of readily absorbed sugars (23). In a recent study involving 59,334 Danish pregnant women, it has been shown that daily intake of artificially sweetened soft drinks may increase the risk of preterm delivery (24). Therefore, it appears that the decrease noted in this study may protect from preterm labour. Clearly further epidemiological studies are required to confirm these effects.

Paradoxically, a wide range of responses concerning the consumption of fried/fast-food during pregnancy was noted. As mentioned previously, poor nutrition may lead to a range of health problems for mothers, including metabolic syndrome and cancer. Pregnancy results in a state of increased energy demand of approximately 300 kcals/day. In addition, maternal energy metabolism is altered during pregnancy and varies greatly among women. The same women who had increased consumption of fast food had also increased the intake of iron-rich foods and dairy products. However, there is no evidence to suggest that this beneficial intake of calcium and iron counteract poor eating habits. Our findings are comparable to an Australian study of 409 women where a high proportion of pregnant women consumed 2 meals of snacks (fast food/take away) per week (25). This finding may also reflect that new generations appear to give up the traditional Mediterranean dietary pattern, adopting new dietary trends (26). In addition, dietary patterns are influenced by various socio-demographic characteristics. Taking these into consideration it is imperative to develop dietary interventions to prevent undesirable health consequences during pregnancy.

Another factor that affects pregnancy is exercise. Regular physical activity is associated with improved physiological, metabolic and psychological parameters, and with a reduced risk of morbidity and mortality (27). In our study [based on the measure of Paffenbarger *et al*(8)] there was a clear shift towards a sedentary lifestyle during pregnancy. For example, there was an increase in overall inactivity of approximately 15% and an equal decrease in moderate exercise. Regular physical activity during pregnancy has been proved to be beneficial for the mother as well as the foetus. Maternal benefits include improved cardiovascular function, minimal weight gain during pregnancy, decreased musculoskeletal discomfort and mood stability, reduction of GDM and gestational hypertension that may lead to preeclampsia (PE). Benefits for the foetus include reduction of fat mass, reduced effects of maternal stress and advanced neurobehavioural maturation (27).

Pregnancy planning and maternal attitudes towards pregnancy also appear to affect foetal weight. Approximately 87 million unplanned pregnancies occur every year worldwide and there is a link between negative experiences of women with unplanned pregnancies before and after labour. For example, two studies have linked unplanned pregnancies with poor relationships with their spouses, experienced financial and educational difficulties and problems with their professional careers (28,29). Data from our study suggest that there is no mother-foetal coherence in the group of unplanned pregnancies, pregnancies since maternal stress did not predict infant weight in that group. Noteworthy findings included the effects of maternal attitude to pregnancy and pregnancy planning on infant birth weight. A potential interpretation would be that possessing a positive or neutral (accepting) attitude towards the pregnancy buffers or protects against any potential negative effect of planning/not planning the pregnancy on foetal weight. However, women may demonstrate negative attitudes even towards a planned pregnancy, and therefore chronic stress may adversely affect foetal development and weight to a greater extent when compared with subjects with a negative attitude but in an unplanned pregnancy.

Consequently, the future directions for healthcare based on these data should be investigated. With regards to the nutritional status and in view of the global epidemic of sedentary life-style and obesity, we propose that pregnant women should increase their physical activity as a preventative measure against adverse pathologies for the mother as well as the foetus. Further studies with larger sample sizes are required to provide solid evidence of associations between increased physical activity and positive outcomes of labour and delivery. The use of a self-reported method for infections may be of clinical significance, as it is likely to allow obstetricians/midwifes to classify patients in a high or low-risk group for predisposition towards pregnancy complications.

Finally, we have also provided evidence that there is no mother-foetal coherence in the group of unplanned pregnancies. Therefore, raising awareness of the impact of unplanned or unintended pregnancy is key. This may be done by educating the public about social and health issues associated with unintended pregnancy. Unintended pregnancy affects individuals, families and communities. Only by communicating this problem to the public, increasing community and individual understanding about prevention and improving access to necessary services, ensures more positive outcomes for both the mother and the foetus.

## Figures and Tables

**Table I.** Demographic details of the general cohort.

General profile	Percentage
Age (years)	
≤20	7.7
21–30	56.7
31–40	35.6
BMI	
Underweight (<18.5)	13.6
Normal (18.5–24.9)	64.1
Overweight (25–29.9)	12.6
Obese (>30)	9.7
Weight of mother as newborn (g)	
Small (<2,500)	5.1
Normal (2,500–3,800)	85.6
Large (>3,800)	9.3
Born prematurely	
Yes	7.1
No	92.9
Body shape (BP) mostly matches yours	
Apple-shape	8.7
Pear-shape	56.3
Proportionate-shape	35.0
Duration of pregnancy (weeks)	
Term (>37)	43.2
Pre-term (<37)	56.8
Foetal weight (g)	
Small (<2,500)	25.3
Normal (2,500–3,800)	72.8
Large (>3,800)	1.9

BMI, body mass index; BP, before pregnancy.

**Table II.** Details of the immune profile of the participants in our study.

Immune profile	Percentage
How many colds did you get during pregnancy?	
0	68.9
1	6.8
2	21.4
3	1.9
4	1.0
How many infections did you get during pregnancy?	
0	77.9
1	10.6
2	9.6
3	1.9
Do you find it hard to ‘fight’ an infection (e.g. cold)?	
Yes	36.5
No	63.5
Are you prone to thrush or cystitis?	
Yes	16.3
No	83.7
How often did you take antibiotics in the last month?	
None	71.1
Once	21.2
Twice	4.8
More than 3 times	2.9
Do you have an inflammatory disease (e.g. arthritis)?	
Yes	6.8
No	93.2
Do you suffer from hay fever, allergies?	
Yes	2.9
No	97.1

**Table III.** Details of the exercise profile of the participants in our study.

Exercise profile	BP (%)	DP (%)
How many flights of stairs do you climb each day (10 steps=one flight)?		
Inactive (0–1 flight of stairs)	23.1	31.7
Light (2–4 flights of stairs)	47.1	47.1
Moderate (5–7 flights of stairs)	29.8	21.2
Active (8+ flights of stairs)	0.0	0.0
How many city blocks do you walk each day (1 block=130 m)?		
Inactive (0–1 city blocks)	12.5	23.1
Light (2–4 city blocks)	34.6	42.3
Moderate (5–7 city blocks)	31.7	22.1
Active (8+ city blocks)	21.2	12.5
How many h/week do you participate in any light sports (e.g. dancing, gardening, walking)?		
Inactive (0–1.5 h/week)	41.3	59.6
Light (1.6–2.5 h/week)	22.1	28.8
Moderate (2.6–3.5 h/week)	28.8	8.7
Active (3.6+ h/week)	7.7	1.9
How many h/week do you participate in any strenuous sports (e.g. running, cycling, swimming, tennis)?		
Inactive (0–1.5 h/week)	76.9	93.3
Light (1.6–2.5 h/week)	8.7	6.7
Moderate (2.6–3.5 h/week)	12.5	0.0
Active (3.6+ h/week)	1.9	0.0

BP, before pregnancy; DP, during pregnancy.

**Table IV.** Details of the nutritional profile of the participants in our study.

Nutrition profile	BP (%)	DP (%)
Are you vegetarian?		
Yes	7.8	7.8
No	92.2	92.2
How often do you buy full-fat dairy products?		
Often	63.1	N/A
Rarely	36.9	N/A
How many meals per week would include any of the following: pies, pastries, fried foods?		
None (0)	1.0	1.0
Light consumption (1–2)	33.0	46.1
Moderate consumption (3–4)	31.1	40.2
Excess consumption (≥5)	35.0	12.7
How many servings of vegetables/legumes do you have each day?		
None (0)	3.9	2.9
Light consumption (1–2)	38.8	29.4
Moderate consumption (3–4)	52.4	60.8
Excess consumption (≥5)	4.9	6.9
How many servings of fruit do you have each day?		
None (0)	1.9	1.0
Light consumption (1–2)	32.1	17.6
Moderate consumption (3–4)	50.5	65.7
Excess consumption (≥5)	15.5	15.7
How many servings of cereals do you have each day?		
None (0)	2.9	2.0
Light consumption (1–2)	1.9	1.0
Moderate consumption (3–4)	33.1	32.6
Excess consumption (≥5)	62.1	64.4
Do you eat iron-rich foods (e.g. lean red meat, chicken, green leafy vegetables) every day?^a^		
Yes	66.7	94.1
No	33.3	5.9
Do you eat ≥2 servings of cheese, milk, yoghurt or calcium-enriched milk every day?^b^		
Yes	65.0	85.3
No	35.0	14.7
How much water/sugar-free drinks do you drink each day?		
<½ liter	5.8	3.0
½ to 1 liter	8.8	4.0
>1 liter	85.4	93.0
How many cups of coffee, black tea or caffeine-containing beverages do you drink each day?^a^		
4–6	1.0	0.0
3–4	27.2	2.0
2–3	43.6	12.7
≤1	28.2	85.3
How many soda, sugary drinks do you normally have each day?^a^		
>3	36.9	2.0
2–3	38.8	29.4
≤1	24.3	68.6
How many alcoholic beverages do you consume on a weekly basis?^a^		
>5	0.0	0.0
3–4	14.6	1.0
≤2	23.3	5.2
None	62.1	93.8
How many times a week do you eat fast food?		
Never	36.9	58.8
1–2 times	42.8	35.3
3–4 times	18.4	5.9
≥5 times	1.9	0.0

BP, before pregnancy; DP, during pregnancy. Significant responses presented in graph format in Fig. 1.

a P<0.0001 and

b P<0.05.
